# Estimation of Bounded and Unbounded Trajectories in Diffusion MRI

**DOI:** 10.3389/fnins.2016.00129

**Published:** 2016-03-31

**Authors:** Lipeng Ning, Carl-Fredrik Westin, Yogesh Rathi

**Affiliations:** Harvard Medical School, Brigham and Women's HospitalBoston, MA, USA

**Keywords:** diffusion MRI, autocorrelation function, single-pulsed field gradient, Ornstein-Uhlenbeck model

## Abstract

Disentangling the tissue microstructural information from the diffusion magnetic resonance imaging (dMRI) measurements is quite important for extracting brain tissue specific measures. The autocorrelation function of diffusing spins is key for understanding the relation between dMRI signals and the acquisition gradient sequences. In this paper, we demonstrate that the autocorrelation of diffusion in restricted or bounded spaces can be well approximated by exponential functions. To this end, we propose to use the multivariate Ornstein-Uhlenbeck (OU) process to model the matrix-valued exponential autocorrelation function of three-dimensional diffusion processes with bounded trajectories. We present detailed analysis on the relation between the model parameters and the time-dependent apparent axon radius and provide a general model for dMRI signals from the frequency domain perspective. For our experimental setup, we model the diffusion signal as a mixture of two compartments that correspond to diffusing spins with bounded and unbounded trajectories, and analyze the corpus-callosum in an *ex-vivo* data set of a monkey brain.

## 1. Introduction

Diffusion MRI (dMRI) is an important clinical tool for non-invasive investigation of tissue microstructure. It can identify brain tissue abnormalities and provide useful image-based biomarkers for diagnosing several neurological and psychiatric disorders. Since the dMRI signal originates from diffusing water molecules, understanding the diffusion processes of these molecules is pivotal for understanding the underlying tissue layout.

Diffusion tensor imaging (DTI) is a classical method for modeling dMRI signals (Basser et al., 1994), where the probability distribution of the displacements of water molecules, also referred to as the ensemble average propagator (EAP), is assumed to be Gaussian. This assumption is not satisfied in practice due to the restrictions and hindrances from cellular and axonal membranes. To account for this deviation from free diffusion, several methods have been proposed to use non-Gaussian functions for modeling the EAP (Cheng et al., 2010; Merlet et al., 2012; Özarslan et al., 2013; Ning et al., 2015). A different group of methods have been proposed to estimate the time-varying diffusion coefficient of the water displacements (Mitra and Halperin, 1995; Novikov et al., 2014; Burcaw et al., 2015). For example, a bi-exponential model has been used in Niendorf et al. (1996) to fit the dMRI measurements; the kurtosis of the diffusion propagator was estimated in Jensen et al. (2005) for investigating the non-Gaussianity of the EAP; and the time-varying feature of the covariance was shown to be closely related to the microstructural arrangement of axons in brain tissue (Burcaw et al., 2015). These methods can provide novel information about the underlying diffusion processes and useful indices for identifying tissue abnormalities.

One way to glean information about the tissue composition is to use the multi-compartment model proposed in Assaf et al. (2008). Very similar to the multi-exponential model, the multi-compartment model also consists of two or more terms that represent the dMRI signal from different parts of the tissue. In particular, the model for the intra-axonal dMRI signal is usually obtained by solving the diffusion equation with suitable boundary conditions (Murday and Cotts, 1968; Neuman, 1974; Stepišnik, 1993), while the extra-axonal compartment is usually modeled by free anisotropic diffusion. Thus, the multi-compartment model provides insights on understanding the reasons for the non-Gaussianity of the EAP and the time-varying feature of the mean-squared displacements. The multi-compartment model was used in Alexander et al. (2010) and Huang et al. (2015) to estimate the axon diameter from *in-vivo* human brain data. However, the estimated axon radius in these works in the monkey corpus callosum was about 5μm, which is much larger than the results obtained from histology studies (Aboitiz et al., 1992; Liewald et al., 2014). Moreover, it was shown in Huang et al. (2015) that the estimated radius was a function of the diffusion time, which is not biologically plausible.

One possibility for the over-estimation and time-dependence of axon diameters could be due to slowly diverging spins in the extra-axonal space (Burcaw et al., 2015). In this approach, it was assumed that the mean-squared displacement of the intra-axonal spins reach a stationary value in very short time, but the time-varying diffusivity from the diverging spins can be misinterpreted as a component from the intra-axonal space, leading to over-estimation and time-dependence of the apparent axon radius. However, the time-dependence of the diffusivity used in Burcaw et al. (2015) was is the long time limit, i.e., ≥100ms, but the diffusion time used in Alexander et al. (2010) was much shorter, i.e., ≤ 50ms. In this relatively shorter time scale, one may be interested to know if it is still reasonable to assume that the mean-squared displacement of bounded diffusion trajectories has reached its stationary value, especially when these trajectories have large radii, e.g., 5μm. If not, what is the relation between the large radii of the bounded components and the time-dependence of the apparent axon radius? The answers to these questions can be obtained via analyzing the dMRI signal from restricted or bounded pores (spaces). In this paper, we demonstrate that the autocorrelation function for diffusion trajectories in restricted pores can be well approximated by an exponential function. Consequently, the multivariate Ornstein-Uhlenbeck (OU) process (Uhlenbeck and Ornstein, 1930) can be applied to model the diffusion of spins in the three-dimensional space with statistically bounded trajectories. The multivariate OU model leads to a matrix-valued autocorrelation function, which can be applied for modeling dMRI measurements acquired using single pulse field gradient or other general type of gradient sequences. Using dMRI data from an *ex-vivo* monkey brain, we show that the proposed model provides a much more accurate fit of the measured dMRI signals, compared to the fits shown for the same data set in Alexander et al. (2010) and Huang et al. (2015). We conjecture that, if the underlying structure contains diffusing spins with bounded trajectories of large radii, then this may possibly explain the overestimation and time-dependence of the estimated axon diameter.

## 2. Theory

The NMR precession frequency of a water molecule at location *x* is given by −γ*g*(*t*) · *x* where γ is the gyromagnetic ratio and *g*(*t*) is a time-varying magnetic field gradient. Let *x*(*t*) represent the trajectory traced by a water molecule (spin) as a function of time *t*. Then the time-dependent phase change for a trajectory *x*(*t*) over the interval [0, *T*] is given by $\phi \left(g\right)=-\gamma \int_{0}^{T}g\left(t\right)\cdot x\left(t\right)dt$. Let $\bar{x}$ denote the center of mass of a trajectory, i.e., $\bar{x}=\frac{1}{T}\int_{0}^{T}x\left(t\right)dt$. Let $\tilde{x}\left(t\right)=x\left(t\right)-\bar{x}$ denote the centered spin trajectory. Then,

$$\phi \left(g\right)=-\gamma \int_{0}^{T}g\left(t\right)\cdot \tilde{x}\left(t\right)dt+\bar{x}\cdot \int_{0}^{T}g\left(t\right)dt.$$

In order to obtain the spin echo, the gradient *g*(*t*) must satisfy $\int_{0}^{T}g\left(t\right)dt=0$. Hence, ϕ(*g*) only depends on the zero-mean trajectory $\tilde{x}\left(t\right)$ with $\phi \left(g\right)=-\gamma \int_{0}^{T}g\left(t\right)\cdot \tilde{x}\left(t\right)dt$. For simplicity of notation, we will use *x*(*t*) for $\tilde{x}\left(t\right)$ as a zero-mean trajectory.

The normalized diffusion signal *E*(*g*) is the ensemble average of all the molecules and is given by $E\left(g\right)=\bar{\exp\left(i\phi \left(g\right)\right)}$ where $\bar{\cdot }$ denotes the ensemble average. Assuming ϕ(*g*) follows a zero-mean Gaussian distribution, i.e., assuming a Gaussian phase approximation, the diffusion signal is given by $E\left(g\right)=\exp\left(-\frac{1}{2}\bar{\phi {\left(g\right)}^{2}}\right)$, where $\bar{\phi {\left(g\right)}^{2}}$ denotes the covariance of ϕ(*g*). Then, the diffusion signal can be rewritten as:

(1) $$\begin{array}{l} E\left(g\right)=\exp\left(-\frac{1}{2}\gamma^{2}\int_{0}^{T}\int_{0}^{T}g\left(t\right)\prime \bar{x\left(t\right)x\left(s\right)\prime }g\left(s\right)dsdt\right), \end{array}$$

where′ denotes the transpose of a vector (or a matrix). Clearly, the positional autocorrelation function $C\left(t,s\right):=\bar{x\left(t\right)x\left(s\right)\prime }$ is key to modeling the dMRI signal.

Consider a sPFG experiment with the pulse width and diffusion time denoted by δ and Δ, respectively. Let *y*_1_, *y*_2_ denote the center of masses (COMs) during the two pulses (Mitra and Halperin, 1995):

(2) $$\begin{array}{l} y_{1}=\frac{1}{\delta }\int_{0}^{\delta }x\left(t\right)dt,\;y_{2}=\frac{1}{\delta }\int_{\Delta }^{\Delta +\delta }x\left(t\right)dt. \end{array}$$

Then the dMRI signal in Equation (1) can be written as:

(3) $$\begin{array}{r} E\left(q\right)=e^{-\frac{1}{2}q\prime R\left(\delta ,\Delta \right)q}, \end{array}$$

where $R\left(\delta ,\Delta \right)=\bar{\left(y_{2}-y_{1}\right)\left(y_{2}-y_{1}\right)\prime }$ denotes the mean-squared displacement of the COMs, and *q*: = γ*gδn* with *g* being the gradient strength and *n* being a unit vector along the gradient direction. Thus, the expression for *R*(δ, Δ) depends on the position autocorrelation function of the water molecules.

### 2.1. On the positional autocorrelation function

For freely diffusing water molecules, the increment of the diffusion process *x*(*s*) − *x*(*t*) for *s* > *t* is uncorrelated with *x*(*t*). The position autocorrelation function is then given by:

$$C_{D}\left(t,s\right)=\bar{x\left(t\right)x\left(s\right)\prime }=2D\min\left(t,s\right),\forall t,s\geq 0,$$

where *D* denotes the diffusivity tensor. The mean-squared displacement *R*_*D*_(δ, Δ) can be explicitly computed as:

(4) $$\begin{array}{l} R_{D}\left(\delta ,\Delta \right)=2D\left(\Delta -\delta /3\right), \end{array}$$

which turns (Equation 3) into the standard diffusion tensor model. Due to restrictions and hindrances, the water molecules in brain tissue are not free to diffuse in all directions. As a result, *R*_*D*_(δ, Δ) does not correctly model the time-varying features of the mean-squared displacements of COMs. Moreover, the diffusion tensor model, while being sensitive, does not provide any specific information about the tissue structure.

In the case for restricted and impermeable pores the expression for the diffusion signal has been derived from the solution of diffusion equation with suitable boundary conditions (Stejskal and Tanner, 1965; Murday and Cotts, 1968; Neuman, 1974; Vangelderen et al., 1994; Åslund and Topgaard, 2009). In particular, the position autocorrelation function along the perpendicular direction of the restricted wall is given by Stepišnik (1993):

(5) $$\begin{array}{l} c_{r⊥}\left(t,s\right)=2\overset{\infty }{\underset{m=1}{\sum }}\frac{r^{2}}{\alpha_{m}^{2}\left(\alpha_{m}^{2}+1-n\right)}e^{-\frac{\alpha_{m}^{2}d_{⊥}}{r^{2}}|t-s|}, \end{array}$$

where *n* denotes the number of restricted dimensions (plane: *n* = 1, cylinder *n* = 2, sphere: *n* = 3), and α_*m*_ is the m-th root of *J*_*n*/2_(α) − α*J*_1+*n*/2_(α) = 0, with *J*_*v*_ being the *v*-th order Bessel function of the first kind, *d*_⊥_ denotes the diffusivity along the perpendicular direction of the restricted walls. The mean-squared displacement of COMs is given by Neuman (1974):

$$\frac{4}{\delta^{2}}\overset{\infty }{\underset{m=1}{\sum }}\frac{r^{2}}{\alpha_{m}^{2}\left(\alpha_{m}^{2}+1-n\right)}f\left(\frac{\alpha_{m}^{2}d_{⊥}}{r^{2}},\delta ,\Delta \right),$$

where the function *f*(·, ·, ·) is defined as

(6) $$\begin{array}{l} f(a,\delta ,\Delta )=(2e^{-a\delta }+2e^{-a\Delta }-e^{-a(\Delta +\delta )}-e^{-a(\Delta -\delta )}\\ \;-2+2a\delta )a^{-2}. \end{array}$$

**Figure 1:**
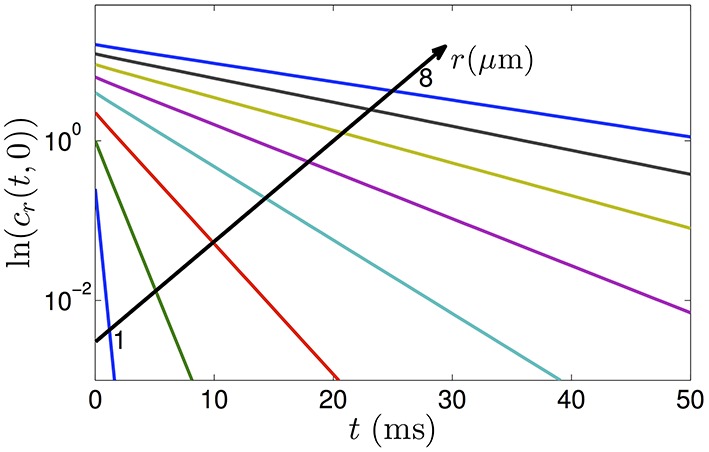
**The logarithm of the positional autocorrelation function of diffusing particles in cylinders with radii varying from 1 to 8 μ*m***. The plots show that the autocorrelation functions decrease approximately as exponential functions of diffusion time.

We note that the autocorrelation function in Equation (5) is an infinite series of weighted exponential functions. As *m* increases, the weighting coefficients get smaller and the exponential function decays quickly. As a result, for long-time scales, the correlation is dominated by the first term corresponding to α_1_. For example, the first three values of α_*m*_ are approximately given by α_1_ = 1.84, α_2_ = 5.33 and α_3_ = 8.54. Assuming *r* = 1μm, *D* = 1μm^2^/ms, *t* = 0.1 ms and *n* = 2, the first three terms of Equation (5) are given by 0.1759, 0.0013 and 5.63 × 10^−6^. Thus, the contribution of the terms for *m* ≥ 2 is very minimal. To show that the correlation function is approximately an exponential function, we plot the logarithm of *c*_*r*_(*t*, 0) with *r* = 1, 2, …8μ*m* in Figure 1 where we include the first 500 terms (*m* = 1, …, 500) in the sum in Equation (5). The linear curves imply that the correlation function of the diffusion process in restricted pores can very well be approximated by a single exponential function, as obtained from our analysis in Equation (13). The validation of the exponential form of the autocorrelation function has also been discussed in Sheltraw and Kenkre (1996) and Burcaw et al. (2015).

In the multi-compartment model used in Alexander et al. (2010), the spins were assumed to be freely diffusing along the direction of the fiber bundles, leading to larger errors in data fitting (see e.g., Figure 10 in Alexander et al., 2010). The large fitting error may be due to the restricted diffusion along the fiber orientation due to axonal undulation as well dispersion. Thus, the intra-axonal spins experience bounded diffusion in a three-dimensional space (and not just in the direction orthogonal to the fiber orientation). For a three-dimensional bounded diffusion process, the corresponding autocorrelation is approximately given by a matrix-valued exponential function. For this reason, we propose to model the three-dimensional bounded diffusion using a multivariate Ornstein-Uhlenbeck process, which provides the expected matrix-valued exponential autocorrelation and can be applied to analyze dMRI signals acquired using any type of gradient sequences.

### 2.2. The Ornstein-Uhlenbeck model

We consider the following multivariate Ornstein-Uhlenbeck (OU) model:

(7) $$\begin{array}{l} \frac{dx\left(t\right)}{dt}=-Ax\left(t\right)+\sqrt{2D}w\left(t\right) \end{array}$$

for the diffusion processes of water molecules with statistically bounded trajectories, where *w*(*t*) denotes the three-dimensional continuous Gaussian noise which is formally considered as the derivative of the Brownian motion with $\bar{w\left(t\right)w\left(s\right)\prime }=\delta \left(t-s\right)Idt$, *A* ∈ ℝ^3 × 3^ is a matrix with all its eigenvalues having positive real parts and the tensor *D* is positive definite and $\sqrt{D}$ denotes the unique positive-definite square root of *D*. This model is a multivariate extension of the one used in Stejskal (1965) and Sevilla and Kenkre (2007). An intuitive interpretation for the model parameter *A* is that it models the rate at which a particle forgets its previous location. As a solution to Equation (7), *x*(*t*) relates to *x*(*s*) with *s* < *t* by,

(8) $$\begin{array}{l} x\left(t\right)=e^{-A\left(t-s\right)}x\left(s\right)+\int_{s}^{t}e^{-A\left(t-\tau \right)}\sqrt{2D}w\left(\tau \right)d\tau , \end{array}$$

where *e*^−*At*^ denotes the matrix exponential function. Clearly, the particle's memory of its previous location decays as an exponential function of time. In anisotropic pores, this rate of “forgetfulness" may be different along different directions and is captured by the eigenvalues of *A*. In the stationary case, *x*(*t*) follows a zero-mean Gaussian distribution with covariance *C* = 〈*x*(*t*)*x*(*t*)′〉 where 〈·〉 denotes the time average of the process. Since the process is also ergodic, the ensemble average of the trajectories of many particles (spins) is the same as their time average. Hence, we also write $C=\bar{x\left(t\right)x\left(t\right)\prime }$.

For the model in Equation (7), the covariance *C*(*t*) is related to the model parameters by the following Lyapunov equation:

(9) $$\begin{array}{l} \frac{dC\left(t\right)}{dt}=-AC\left(t\right)-C\left(t\right)A\prime +2D. \end{array}$$

Assuming that the covariance *C* is stationary, leads to the following equality:

(10) $$\begin{array}{l} -AC-CA\prime +2D=0. \end{array}$$

For a positive-definite tensor *D* and a matrix *A* with eigenvalues having positive real parts, the covariance *C* can be uniquely determined by solving the linear system of equations in (10). On the other hand, we note that there may be different pairs of *A, D* that give the same *C*. Throughout this paper, we assume that *A, C, D* are invertible. Next, we show that the time-reversibility of the of process imposes structural constraints on the parameters *A* and *D*, which in turn reduces the number of free variables used in the model.

Let $C_{\text{ou}}\left(t,s\right):=\bar{x\left(t\right)x\left(s\right)\prime }$ denote the position autocorrelation function with *C*_ou_(*t, t*) = *C*. From the relation between *x*(*t*) and *x*(*s*) given by Equation (8), we obtain

$$\begin{array}{l} C_{\text{ou}}(t,s)=\{\begin{array}{ll} e^{-A(t-s)}C & \;\text{if}\;t\geq s,\\ Ce^{-A\prime (s-t)} & \;\text{if}\;t<s, \end{array} \end{array}$$

where we have used the fact that *w*(τ) is uncorrelated with *x*(*s*) for τ ≥ *s*. Moreover, we assume that the diffusion process is time reversible since the trajectories of diffusing molecules should have the same pattern during both the forward and backward evolution of time. This assumption implies that the joint probability distribution function of [*x*(*t*)′*x*(*s*)′]′ is the same as that of [*x*(*s*)′*x*(*t*)′]′. It further implies the following symmetry:

(11) $$\begin{array}{l} C_{\text{ou}}\left(t,s\right)=C_{\text{ou}}\left(s,t\right). \end{array}$$

In particular, setting *s* = 0 leads to *e*^−*At*^*C* = *Ce*^−*A*^′*t* for any *t* ≥ 0. Taking the derivative of both sides at *t* = 0, we obtain: *AC* = *CA*′. Substituting this relation in Equation (10), we obtain:

(12) $$\begin{array}{l} AC=D. \end{array}$$

In the original model (Equation 7), the matrix *A* had 9 variables and the tensor *D* had 6. However, following Equation (12), *A* can be written as *A* = *DC*^−1^. A consequence of this relation is that the eigenvalues of the matrix *A* become real-valued with total number of free model parameters reducing to 12. Further, one can also assume that *A, C* and *D* have the same eigenvectors, which leads to a simplified model with 9 parameters. If Equation (12) holds, we denote the autocorrelation function as:

(13) $$\begin{array}{l} C_{\text{ou}}\left(t,s\right)=e^{-A|t-s|}C \end{array}$$

which is the same as *Ce*^−*A*^′|*t*−*s*|. From Equations (13) and (11), we can derive that

$$\begin{array}{l} \bar{y_{1}y_{1}^{\prime }}=\frac{2}{\delta^{2}}\left(e^{-A\delta }-I+A\delta \right)A^{-2}C\;,\\ \bar{y_{2}y_{1}^{\prime }}=\frac{1}{\delta^{2}}\left(e^{-A(\Delta +\delta )}+e^{-A(\Delta -\delta )}-2e^{-A\Delta }\right)A^{-2}C. \end{array}$$

Further, $\bar{y_{2}y_{2}^{\prime }}=\bar{y_{1}y_{1}^{\prime }}$ and $\bar{y_{1}y_{2}^{\prime }}=\bar{y_{2}y_{1}^{\prime }}$. By substituting these expressions for $\bar{y_{i}y_{j}^{\prime }}$ with *i, j* = 1, 2, we obtain the expression for the mean-squared displacement between the COMs as:

(14) $$\begin{array}{l} R_{\text{ou}}\left(\delta ,\Delta \right)=\frac{2}{\delta^{2}}f\left(A,\delta ,\Delta \right)C, \end{array}$$

where the function *f*(·, ·, ·) is defined as in Equation (6).

The model parameter *A* characterizes the rate at which a particle forgets its previous location. If the eigenvalues of *A* are small, then the diffusion signal in Equation (3) is similar to free-diffusion. To show this, we let *A* = ϵ*A*_0_ for a constant *A*_0_. Then, it is straightforward to show that

$$\underset{\epsilon \to 0}{\lim}\frac{2}{\delta^{2}}f\left(\epsilon A_{0},\delta ,\Delta \right)\left(\epsilon^{-1}A_{0}^{-1}D\right)=2D\left(\Delta -\delta /3\right)=R_{D}\left(\delta ,\Delta \right).$$

Thus, free and hindered diffusion processes can be considered as a special case of an OU diffusion process in the limiting case of very small attraction potential.

### 2.3. A frequency-domain model for dMRI signal

The OU model can also be studied from a frequency domain perspective allowing for any type of gradient sequences, such as the multiple-pulsed field gradient (mPFG) (Avram et al., 2013) and oscillating gradient waveforms (Gore et al., 2010). Let *g*(*t*) denote the gradient waveform and let $G\left(\omega \right):=\overset{T}{\underset{0}{\int }}g\left(t\right)e^{-i\omega t}dt$ denote its Fourier transform. We denote by *X*(ω) the power spectral density (PSD) of the OU process which is defined by the equation

(15) $$\begin{array}{l} C_{\text{ou}}\left(t,s\right)=\frac{1}{2\pi }\int_{-\infty }^{\infty }X\left(\omega \right)e^{-i\omega |t-s|}d\omega \end{array}$$

and is given by

$$X\left(\omega \right)=2{\left(i\omega I+A\right)}^{-1}D{\left(i\omega I+A\right)}^{-*}.$$

Then the dMRI signal from the molecules with bounded trajectories is given by

(16) $$\begin{array}{l} E\left(g\right)=\exp\left(-\frac{1}{4\pi }\gamma^{2}\int_{-\infty }^{\infty }G{\left(\omega \right)}^{*}X\left(\omega \right)G\left(\omega \right)d\omega \right). \end{array}$$

We note an important difference between the above formulation and the frequency-domain expression used in Stepišnik (1993) and Gore et al. (2010). The expression in Equation (16) is derived for modeling dMRI signals from spins with bounded diffusion trajectories and is written in terms of the position PSD *X*(ω) and the Fourier transform of the gradient sequence. In the sPFG case, the expression in Equation (16) will be identical to the one obtained earlier in Equation (3). On the other hand, the frequency-domain expression in Stepišnik (1993) and Gore et al. (2010) was written in terms of the PSD of the velocity process and the Fourier transform of the integral of the gradient sequence.

### 2.4. On the time-dependence of the apparent axon radius

The intra-axonal and extra-axonal dMRI signals have different time-domain behaviors, making it possible to decompose the two components from dMRI measurements acquired using different diffusion times. However, the experimental results obtained in Huang et al. (2015) show that the estimated axon size also varies with diffusion time, which is biologically not possible. Understanding the mean-squared displacements of the bounded and unbounded components could provide more insights into understanding the time-dependence of the estimated axon radius in these works.

In the narrow-pulse case, the mean-squared displacement between the COMs from the OU model is given by $\underset{\delta \to 0}{\lim}R_{\text{ou},⊥}\left(\delta ,\Delta \right)=2c_{⊥}\left(1-e^{-a_{⊥}\Delta }\right)$, where *c*_⊥_, *a*_⊥_ denote the eigenvalue of *C* and *A* along the perpendicular direction to the orientation of the fiber bundles. It is usually assumed that the diffusion time Δ is long enough so that the mean-squared displacement reaches its long-time limit 2*c*_⊥_. On the other hand, the long-time limit of the mean-squared displacement in an isotropic cylinder is given by *r*^2^/2 where *r* denotes the cylinder radius. By equating the two expressions for mean-squared displacements, we see that the apparent axon radius is a function of time, and is given by:

(17) $$\begin{array}{l} r_{\text{app}}^{2}\left(\Delta \right)=4c_{⊥}\left(1-e^{-a_{⊥}\Delta }\right), \end{array}$$

which is a monotonic increasing function of Δ and converges to a constant 4*c*_⊥_ at long diffusion time. From the analysis in Section 2.1, the exponent *a*_⊥_ is approximately given by $a_{⊥}\approx \frac{\alpha_{1}^{2}d_{⊥}}{r^{2}}$. Thus, if the underlying structure contains bounded spaces with radii being about 5μm and if the diffusivity is about 1μm^2^/ms, then it takes about 20 ~ 30ms for the mean-squared displacement to reach its limiting value. Thus, the time dependence of the apparent axon radius at short-time scale may be due to the slowly decaying autocorrelation function from diffusing spins with bounded trajectories of large radii.

On the other hand, at long-time scale, the time-dependence of the apparent axon radius may be due to the slowly diverging spins in the extra-axonal space. It was pointed out in Burcaw et al. (2015) that the apparent diffusivity monotonically decreases with increasing diffusion time at long-time scale, e.g., Δ ≥ 100ms. If the time-dependent terms of the diffusivity is misinterpreted as a component from the intra-axonal space, then the apparent axon radius is given by Burcaw et al. (2015):

(18) $$\begin{array}{l} r_{\text{app}}^{2}\left(\Delta \right)=\langle r^{4}\rangle /\langle r^{2}\rangle +\text{const.}\ln\left(\Delta /t_{c}\right), \end{array}$$

where 〈*r*^2^〉 and 〈*r*^4^〉 are the second and the fourth order moments of the axon radius distribution. The ratio 〈*r*^4^〉/〈*r*^2^〉 is the expected value of the squared apparent axon radius in the absence of extra-axonal space. Thus, the time-dependent diffusivity in the extra-axonal space will also lead to an over-estimation of the apparent axon radius. Finally, we also remark that the non-Gaussianity of the extra-axonal diffusion propagator should also be taken into account at long-time scale, since it may also lead to biased estimation of the diffusivity and the axon radius.

### 2.5. The multi-component signal model

Assuming that the diffusion process in brain tissue can be decomposed into two categories that have bounded and unbounded trajectories, we can denote the dMRI signal using the following form:

(19) $$\begin{array}{l} E\left(q\right)=pE_{\text{bounded}}\left(q\right)+\left(1-p\right)E_{\text{unbounded}}\left(q\right), \end{array}$$

where *p* and 1 − *p* denote the relative volume fractions of the molecules with bounded and unbounded trajectories, respectively. For dMRI signal from sPFG experiments, the signal model is given by:

(20) $$\begin{array}{l} E(q)=pe^{-\frac{1}{2}q\prime R_{\text{ou}}(\delta ,\Delta )q}+(1-p)e^{-\frac{1}{2}q\prime R_{D}(\delta ,\Delta )q}, \end{array}$$

where *R*_*D*_ and *R*_ou_ are defined in Equations (4) and (14), respectively. The main difference between Equation (20) and the model used in Alexander et al. (2010) and Huang et al. (2015) is that the bounded component is given by a multivariate OU model, which assumes that the diffusing spins along the fiber-bundle direction also have bounded trajectories.

## 3. Experiments

We used the proposed model to analyze an *ex-vivo* data set from a young adult female vervet monkey brain. The data set was obtained from the Danish Research Center For Magnetic Resonance (http://dig.drcmr.dk/activeax-dataset/), with scanning done as described in Dyrby et al. (2010). The data was acquired on a 4.7 T Varian scanner with the following acquisition parameters consisting of three sPFG experiments: δ = {10, 7, 17}ms, Δ = {16, 45, 35}ms and *g* = {0.14, 0.13, 0.14}T/m respectively. The *b*-values for the three experiments were 1900, 3100, 13000*s*/*mm*^2^, and the *q*-values were 0.37, 0.24, 0.64/μm, respectively. Each acquisition consisted of 90 gradient directions with a spatial resolution of 0.4 × 0.4 × 0.5mm^3^ and TE/TR = 60/5000ms.

We used the finite-pulsed model of Equation (20) for fitting the dMRI measurements in order to estimate the volume fraction of the molecules with bounded trajectories and the radius of these trajectories. We assume that the tensors *A, C* in the mean-squared displacement *R*_ou_(δ, Δ) and the tensor *D* in *R*_*D*_(δ, Δ) have cylindrical symmetry with the same set of eigenvectors. Then the total number of variables in our model (Equation 20) is 9 (2 eigenvalues for each of the tensors *A, C, D*_0_, the weight *p* and the principal diffusion direction *n*). Although the diffusion process along any radial direction in the cross-sectional plane can be characterized by a one-dimensional model, the three-dimensional Ornstein-Uhlenbeck process is necessary for modeling diffusion in the three-dimensional space given that the orientation of the fiber-bundle is not known *a-priori*.

In each voxel of a selected region of the corpus-callosum, we used a nonlinear least-squares method (via Matlab command *lsqnonlin*) to estimate the 9 model parameters from a total of 270 measurements. The computational time was about 4 h for the entire data set using a 12-core workstation. We note that Equation (20) is a highly nonlinear function of the variables. In order to restrict the space of possible solutions to a biologically meaningful range, we applied suitable constraints to the model parameters based on prior knowledge about the tissue. For example, the autocorrelation function of the OU process should decay fast enough so that *R*_ou_(δ, Δ) can be distinguished from *R*_*D*_(δ, Δ). Thus, we assumed that the eigenvalues of *A* to be not smaller than 80*s*^−1^, where the lower-bound is approximately the exponent of position autocorrelation function for molecules in a cylindrical axon with diameter 6μm computed according to Equation (5). We also note that since *R*_ou_(δ, Δ) depends on both *A* and *C*, the constraint on *A* makes the estimated value for *C* more sensitive to subtle variations in the diffusion signal.

## 4. Results

**Figure 2:**
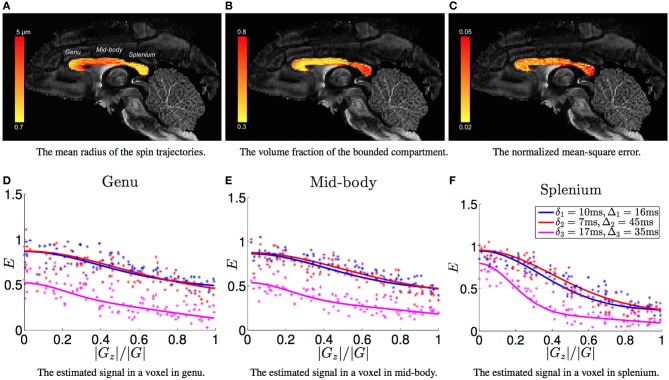
**(A–C)** shows the estimated radius of bounded trajectories, the fraction of the bounded compartment in the dMRI signal and the normalized mean-square error between the estimated signal and the measurements, respectively. **(D–F)** shows the measured (dot points) signal and the estimated signal (solid lines) in three representative voxels from the genu, mid-body and splenium areas, respectively, with the horizontal axis being absolute value of the inner product between the gradient direction and the axon orientation.

For the estimated model parameters in each voxel, let *c*_⊥_ denote the smallest eigenvalue of *C*. Then, we define $\sqrt{c_{⊥}}$ as *the average radius of the bounded trajectories of the diffusing molecules*. Figure 2A shows the estimated average trajectory radius ($\sqrt{c_{⊥}}$) in the corpus-callosum of the monkey brain with the background being the standard fractional anisotropy (FA) image. We can see that the radius in the mid-body of the corpus-callosum is larger than the genu and the splenium. We note that a similar pattern for axon diameters has also been observed from histology analysis in mouse (Barazany et al., 2009), monkey and human brains (Caminiti et al., 2013). We note that the axon radius reported from histology studies of the monkey corpus-callosum range between 0.5−2.5μm, whereas the estimated values of $\sqrt{c_{⊥}}$ are in the range 0.7−5μm. We conjecture that the larger value for the radius of spin trajectories compared with the axon size may be due to bounded diffusion processes with larger radii in densely packed extra axonal space. The estimated $\sqrt{c_{⊥}}$ also reveals a pattern similar to the known distribution of axon sizes as larger axons will create more space for water molecules to diffuse, leading to trajectories with larger radii.

Figure 2B shows the estimated relative volume fraction *p*, which has an inverse contrast compared to Figure 2A. Based on the study of rhesus monkey corpus callosum in Lamantia and Rakic (1990), it was pointed out in Burcaw et al. (2015) that the volume fraction of extra-axonal space is about 0.3. On the other hand, the intra-axonal diameter is about 0.6 times the myelin diameter, i.e., the g-ratio is 0.6 (Rushton, 1951). Thus, the average fraction *p* in corpus callosum should be about 0.3/(0.3+0.7·0.6^2^)≈0.5. The significantly higher values in the splenium area of Figure 2B indicates that there is a large fraction of extra-axonal water molecules having bounded trajectories, which may be caused by very densely packed axons.

Figure 2C shows the normalized mean-square error (NMSE) for the estimated signal with NMSE defined as ||*E* − *Ê*||^2^/||*Ê*||^2^ with *E* and *Ê* being the vector of measured signal and the estimated signal, respectively. In most voxels, the NMSE is around 0.03 implying the proposed model fits the measurement accurately. Figures 2D–F show the estimated signal in three representative voxels using the three acquisition parameters. Note that the fit to the data is quite accurate for all the three acquisitions, which is in contrast to the results reported in Alexander et al. (2010), where the signal for the scan with δ = 17ms was highly overestimated leading to a significant overestimation of the axon diameter (on the same subset of measurements).

**Figure 3:**
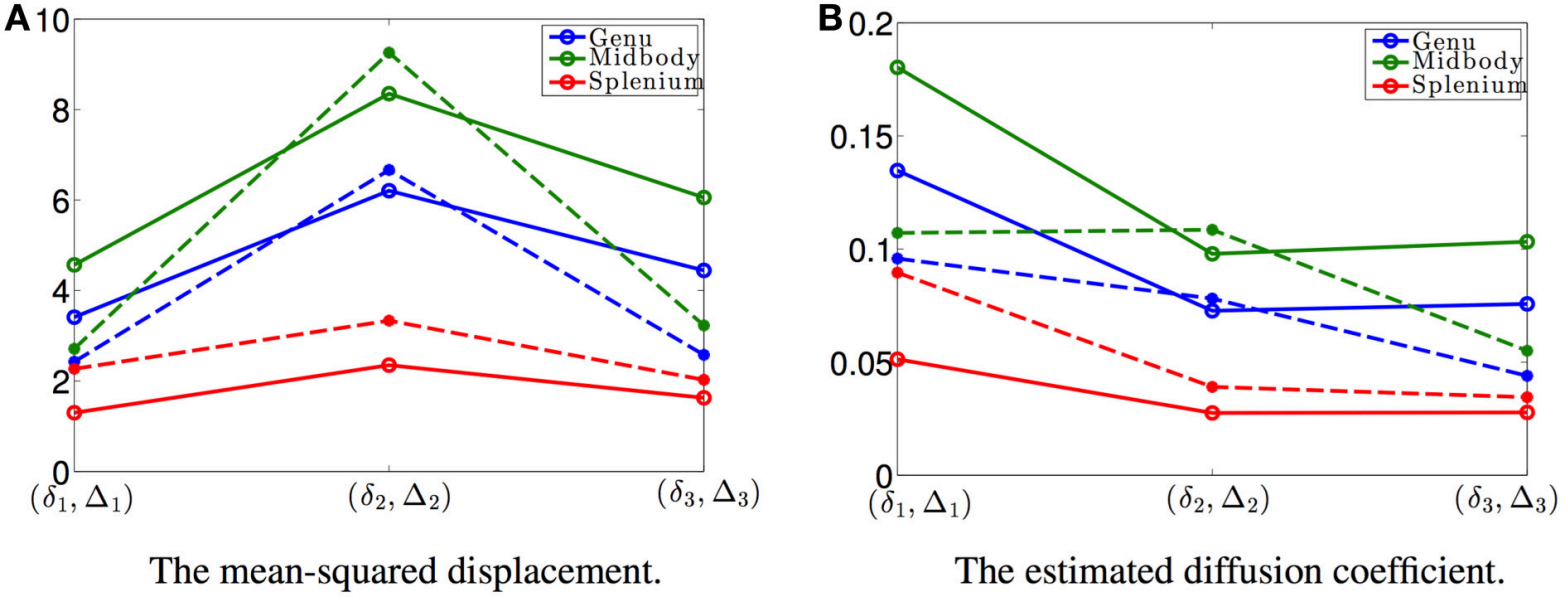
**(A)** shows the mean-squared displacements of the three data sets with different (δ, Δ) with the values shown in Figure 2F: the solid curves are the estimated results using the proposed model and the dashed lines are the corresponding results obtained from the DTI model, respectively. **(B)** shows the corresponding time-dependent diffusion coefficients for the three data sets.

According to Equation (20), the estimated mean-squared displacements of the water molecules are given by *pR*_ou_(δ, Δ) + (1 − *p*)*R*_*D*_(δ, Δ). In order to compare this model with DTI, we also computed the mean-squared displacements using DTI for each data set. The solid and dashed plots in Figure 3A show the estimated mean-squared displacements along the radial direction of the fiber bundles obtained using the proposed model and DTI, where the blue, green and red curves denote the average values from four voxels in the genu, midbody, and splenium areas, respectively. The solid plots show similar features as the dashed lines. Figure 3B shows the corresponding diffusion coefficients estimated using the proposed model and DTI, respectively, with the diffusion coefficients from the proposed model given by (*pR*_ou_(δ, Δ) + (1 − *p*)*R*_*D*_(δ, Δ))/(Δ − δ/3). The reasons for the differences between the results include the measurement noise and that the proposed model (Equation 20) is non-Gaussian, i.e., a non-exponential function of the *b*-value, while DTI corresponds to a Gaussian model. The Gaussian model is not able to correctly estimate the diffusion coefficients at high *b*-values as in the data sets used in this experiment.

## 5. Discussion and conclusion

In this paper, we introduced an approach for modeling the matrix-valued position autocorrelation function for diffusing spins with statistically bounded trajectories using the multivariate Ornstein-Uhlenbeck process. We provided detailed analysis on the relation between the model parameters and the corresponding structure of the bounded space. We also analyzed the relation between the autocorrelation function and the time-dependent axon radius in short-time scale. Moreover, the OU model also provides a simple frequency-domain expression for dMRI signals acquired using any type of gradient sequences.

We applied the proposed method on dMRI data acquired from an *ex-vivo* monkey brain. Our results show that the estimated radius of the bounded trajectories could provide more specific structural information about the tissue compared to the standard measures of fractional anisotropy (FA), despite the fact that the proposed measure is a function of both the axon radius and the volume fraction (axonal packing). Moreover, the results also show that the proposed model better fits the acquired measurements compared with the results shown in Alexander et al. (2010), implying that the diffusion spins along the fiber-bundle direction may also include some bounded component. However, the estimated radii of the bounded trajectories are still larger than the results from histology studies. We conjecture that one possibility for the over-estimation is due to the bounded diffusion trajectories in the extra-axonal space with large radii. We also note that another possible reason for the overestimated radii is due to the variability of the fiber-bundle orientation in the corpus callosum (Nilsson et al., 2012; Ronen et al., 2013). Future work will be on validating the proposed model using more measurements acquired using several measurements with varying diffusion times and on extending the proposed method for characterizing dMRI signal from voxels with fiber crossings.

## Author contributions

LN contribution in the submitted article includes the development of the theory, implementing the numerical simulations and writing the initial version of the manuscript. YR contribution was to provide guidelines and inputs on the development of the theory and to improve the quality of the paper, while CW helped in improving the quality of the manuscript.

### Conflict of interest statement

The authors declare that the research was conducted in the absence of any commercial or financial relationships that could be construed as a potential conflict of interest.
